# Reflections on patient engagement by patient partners: how it can go wrong

**DOI:** 10.1186/s40900-023-00454-1

**Published:** 2023-06-12

**Authors:** Dawn P. Richards, Sabrina Poirier, Vina Mohabir, Laurie Proulx, Sue Robins, Jeffery Smith

**Affiliations:** 1 Canadian Institutes of Health Research Institute of Musculoskeletal Health and Arthritis, University of British Columbia, Vancouver, BC Canada; 2 Five02 Labs Inc., Toronto, ON Canada; 3 Patient Partner, Toronto, ON Canada; 4 Patient Partner for Myalgic Encephalomyelitis Research, Halifax, NS Canada; 5 Child Health Evaluative Sciences, The Hospital for Sick Children, Toronto, ON Canada; 6 Canadian Arthritis Patient Alliance, Ottawa, ON Canada; 7 Patient Partner, Ottawa, ON Canada; 8 Bird Communications, Vancouver, BC Canada; 9 Patient Partner for Myalgic Encephalomyelitis Research, Toronto, ON Canada

**Keywords:** Patient engagement, Family engagement, Patient and public involvement, Power dynamics in healthcare, Power imbalance, Tokenism, Patient partner

## Abstract

As six patient partners in Canada, we aim to contribute to learning and to provide an opportunity to reflect on patient engagement (PE) in research and healthcare environments. Patient engagement refers to “meaningful and active collaboration in governance, priority setting, conducting research and knowledge translation” with patient partners as members of teams, rather than participants in research or clinical care. While much has been written about the benefits of patient engagement, it is important to accurately document and share what we term ‘patient engagement gone wrong.’ These examples have been anonymized and presented as four statements: patient partners as a check mark, unconscious bias towards patient partners, lack of support to fully include patient partners, and lack of recognizing the vulnerability of patient partners. The examples provided are intended to demonstrate that patient engagement gone wrong is more common than discussed openly, and to simply bring this to light. This article is not intending to lay blame, rather to evolve and improve patient engagement initiatives. We ask those who interact with patient partners to reflect so we can all work towards improving patient engagement. Lean into the discomfort with these conversations as that is the only way to change these all too recognizable examples, and which will lead to better project outcomes and experiences for all team members.

## Abstract (plain language summary)

We are six patient partners in Canada who aim to contribute to learning and to provide an opportunity to reflect on patient engagement (PE) in research and healthcare environments. Patient engagement refers to “meaningful and active collaboration in governance, priority setting, conducting research and knowledge translation,” where patient partners are members of the teams, rather than participants in research or those seeking clinical care. It appears more has been written on the benefits rather than the risks of patient engagement and we feel it is important to document and share what we call ‘patient engagement gone wrong.’ We have anonymized these examples and sorted them into four statements: patient partners as a check mark, unconscious bias towards patient partners, lack of support to fully include patient partners, and lack of recognizing the vulnerability of patient partners. These statements and their examples are meant to show that patient engagement gone wrong is more common than discussed openly, and to simply bring this to light. With this commentary, we do not mean to lay blame, and instead wish to evolve and improve patient engagement initiatives. We ask those who interact with patient partners to reflect so we can all work towards improving patient engagement. Lean into the discomfort with these examples, as that is the only way to change these all too recognizable statements, and which will lead to better project outcomes and experiences for all team members.

## Background

This article aims to contribute to the growth and evolution of patient engagement (PE) in research and healthcare, sometimes also called patient and public involvement or PPI. We hope this creates an opportunity for those reading to reflect on the lived experiences of patient partners involved in engagement activities. The term patient partner includes people with their own health issues and experiences and includes caregivers, family members, and friends, who actively contribute to research or quality improvement teams [1]. Patient partners in the context of this article does not refer to participants in research studies or patients seeking clinical care. Further, we use the Canadian Institutes of Health Research’s (CIHR’s) definition of patient engagement in research which is “[m]eaningful and active collaboration in governance, priority setting, conducting research and knowledge translation” [2]. This definition is similar to the National Institute for Health and Care Research’s definition of patient and public involvement in research which is “an active partnership between members of the public and researchers. This means that members of the public work alongside the research team and are actively involved in contributing to the research process as advisers and possibly as co-researchers” [3]. We see the aim of patient engagement being true partnership with patients, where they are equal partners on teams.

Much has been written on the benefits of patient engagement including: facilitating recruitment to and maintaining participation in research studies and clinical trials; leveraging patient partners’ own experiences and insights to provide additional context to goals of and treatments under study; helping knowledge translation (e.g. through making and sharing results that are more relevant and credible to study populations); and potentially even contributing to better and/or different outcomes [4–8]. As the practice of patient engagement continues to evolve and grow, it is equally important to be aware of some of patient engagement’s potential risks or challenges to patients. To name a few, these include the risks of tokenism [9–12], power imbalances and dynamics [10, 13], not having the tools for equitable engagement [7], questioning reasons for engagement [8], lack of accessible and patient-friendly training for patient partners, and a lack of training for other team members [14]. Despite these potential risks and harms due to patient engagement in research and healthcare, patient partners continue to remain involved as: “*It is our lives that are at stake, after all*….”[5]. Many patient partners engage to prevent others from dealing with what they have; to help meet unmet needs of under resourced communities; to develop, and to have a voice in research [15], policy, and clinical care. While much has been written about the benefits and challenges to patient engagement by research teams that may include patient partners, there appears to be little that is written solely from the perspective of patient partners.

We are six patient partners in Canada choosing to bring to light and discuss the difficult situations when patient engagement does not work well for patient partners and may even cause harm. Having done work in Canada and internationally, these experiences are not unique to the Canadian context. These real examples are based on our own experiences, and are presented here in an anonymous, composite way. Anyone is capable of making these mistakes unconsciously. We ask readers to reflect on the examples with a growth mindset, and on their potential to be a partner who lessens the power imbalances that exist in patient engagement. This approach to partnership will change the research team dynamic and may improve the situation and project for all involved. This authorship team has all experienced these examples or variations of them, and we ask readers to think of the patient partners who do not feel safe enough to bring up these experiences. These examples are common and well known within the patient partner community. We provide readers the necessary content to constructively reflect and consider how their own power and privilege may be able to change these scenarios. As a patient engagement community, we are all learning together and are invested in the goals of better research results, outcomes, and partnership, which will result from improving our approaches.

As authors of this paper, we came together as a team after two of us (SP, DPR), brainstormed the original concept for this paper based on our experiences, and realized that others may benefit from them. We invited others to the team based on different experiences and backgrounds (e.g., the conditions a number of us live/lived with, caregiver experiences, location, and other intersectionality-related factors). We have 78 years of combined experience as patient partners in a variety of settings. Yet, even we continue to struggle to be meaningfully engaged from concept initiation or design to dissemination, we have often been the sole patient partner included on a team, and we still have to advocate and educate for full support and accommodations to be on research teams. We are among the patient partners who have had the privilege to be invited and included thus far while many potential patient partners are still excluded from these spaces or opportunities. Much more work is needed in these spaces to increase inclusion and safety for patient partners. Lastly, while we have all had positive experiences in patient engagement, the negative experiences take a deep toll on us. Even with our collective experience these situations leave us wondering about the value we bring or if we should continue given the risks posed to us. We hope to help other patient partners and research teams recognize and mitigate these risks in their own work.

## Main text

### Four statements of patient engagement gone wrong

**Fig. 1:**
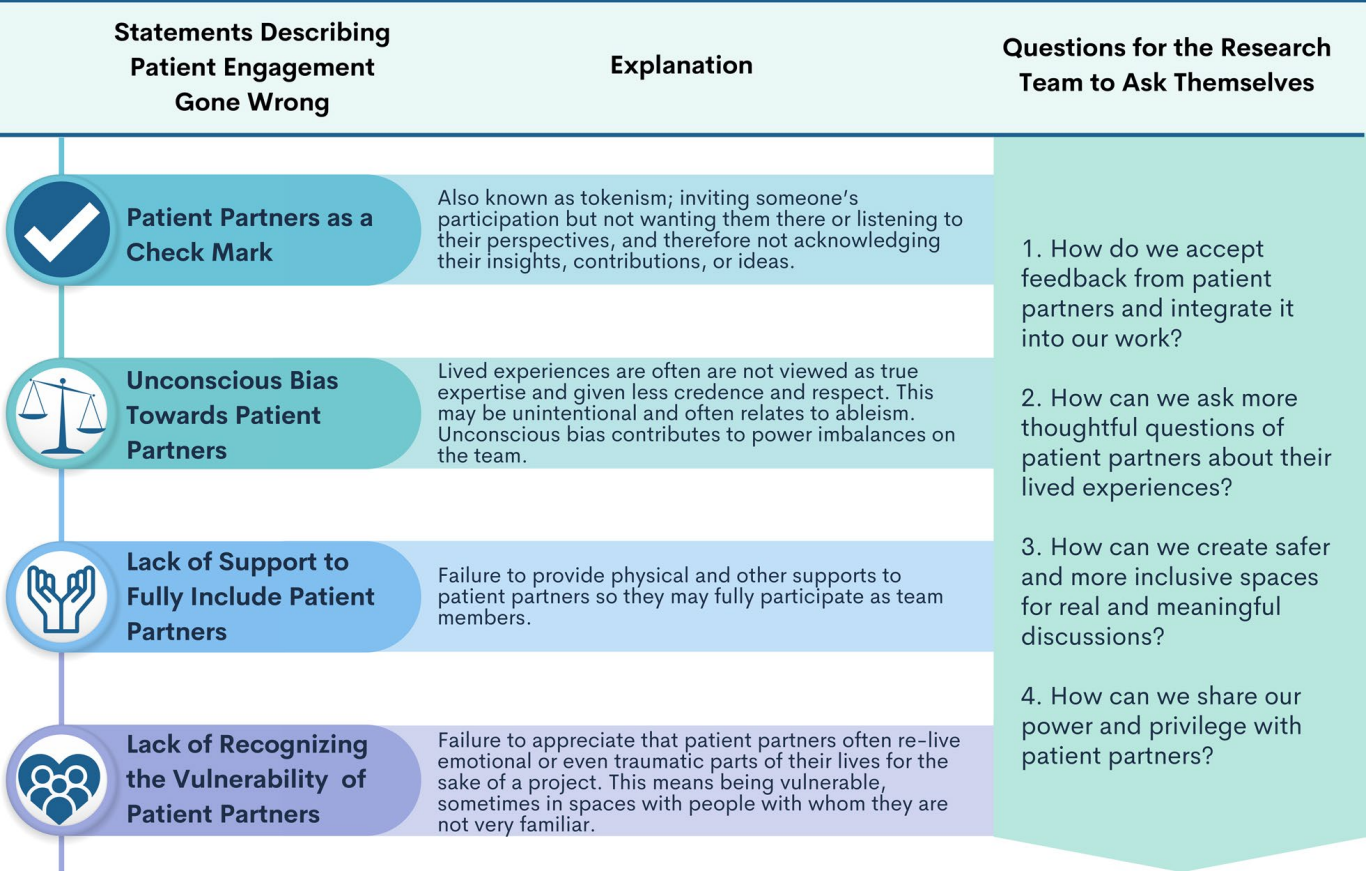
Statements and their explanations of patient engagement gone wrong, along with questions for the research team members to ask about how they could improve or prevent these situations

Below we have identified four problem statements that describe situations in patient engagement that we have all faced (see Fig. 1). We provide anonymous examples for reflection along with how these examples impacted our work, emotions, and even our future work as patient partners. As you read the situations, we ask you to reflect: *Have you seen these situations in your own work? Have you spoken to someone with lived experience? What have you done or would you do to prevent them or to negate these situations? How could you aim to avoid the situation altogether in the future? Who has the power and privilege in this situation?*

**1. Patient partners as a check mark**

This statement can also be called tokenism. Tokenism is essentially inviting someone to participate but not wanting them there or listening to their perspectives and not acknowledging their insights, contributions, or ideas [9–11]. Some real examples in which patient partners may feel tokenized:

- Being invited to grant application teams close to deadline, not being able influence the application, or not being told of the grant competition results.
- Being invited to meetings or conferences that promote but do not embody the #PatientsIncluded designation [16].
- Being co-chairs of meetings or committees without full support to create or influence the agenda or to participate fully.
- Not being invited to speak or share thoughts on a team or at meetings, having feedback and contributions minimized or dismissed, or seeing decisions made when patient partners are absent.
- Being dropped or ignored (i.e., ‘ghosted’[17]) by a team when difficult questions are asked.
- Meetings scheduled when convenient for everyone except patient partners or inviting patient partners to select meetings without providing an opportunity for input on the best approach for them.
- No opportunities for feedback about the engagement experience during the engagement or when it formally ends, or seeing defensive responses to feedback or no effort to understand and acknowledge the patient partner’s experiences.
- Taking credit for working with patient partners while not providing them knowledge translation opportunities (e.g., preparing publications or speaking at conferences).
- Being involved in poorly conducted meetings with little consideration for the importance of introductions and relationship building, discussions, safe spaces, exploring areas of disagreement.
- Being involved or excluded based on age, ability, race, diagnosis, and other components of an intersectional identity.

These situations set patient partners up as an after-thought—not really integral or ‘important enough’ members of the team, and who are meant to agree with what is said at meetings. These situations do not support patient partners bringing up tough questions that might challenge thoughts and beliefs, and in some cases, discourage them from asking for supports that enable full participation. If the team has no other like-minded patient partners or a safe contact for a discussion about the engagement experience, it leaves patient partners feeling gaslit [18], and questioning the validity of their emotions (e.g., being ‘overly sensitive’).

**2. Unconscious bias towards patient partners**

Unconscious bias is defined by the Canadian Institutes of Health Research as “an implicit, unintentional attitude or assumption that affects the way you think and act” [19]. Patient partners on a team are becoming more common, but is not the norm. Research and healthcare can be hierarchal, where patient partners are not as high on that hierarchy as those who have titled, formal credentials. Lived experiences are often given less credence and respect and are not viewed as true expertise. There are often experiences related to ableism, which is defined as “a set of beliefs that guide cultural and institutional practices ascribing negative values to individuals with disabilities whilst deeming able-bodied and able-minded individuals as normal, therefore superior to their disabled counterparts”[20] (which may also be experienced by academics who are disabled [21]). Ableism is made more challenging within the context of intersectionality where other identities, such as sex, gender, and ethnicity, compound to create additional barriers for patient partners. Unconscious bias sets a power dynamic even if unintended, and examples experienced here include:

- The mostly able-bodied and healthy team members not fully understanding patient perspectives, feedback and ideas, given their differences in experiences which may be compounded by a lack of listening or empathy.
- Inviting patient partners who mirror the team’s demographics or who have the privilege and time to be engaged and who do not require additional resources or supports.
- Preconceived judgements or stereotypes that lead to underestimating the capabilities and intellect of patient partners and that fail to recognize that many patient partners have full lives, skills, experiences and education [22].
- Being told that patient partners need a specific graduate level education to undertake certain types of roles or excluding patient partners who have intellectual or developmental disabilities.
- Team members insisting on being called by their formal academic titles.
- Racist and ableist language, and gaslighting patient partners when they share the harm and impact of these words (e.g., dark horse, crazy).
- Unconscious homophobic, transphobic, ableist and racist behaviour is still commonplace when interacting with patient partners who have experienced a large amount of trauma from health systems.

Experiences like these leave patient partners feeling that they are unimportant, not worth the time or effort or resources to be engaged, ‘lesser’ than individuals who are not disabled or who do not identify as patients, and even not as smart as other team members. Patient partners often deal with unconscious bias in the healthcare system and in their everyday lives so having these biases reinforced is demoralizing and leaves patient partners questioning their motivations. Patient partners can have traumatic health care experiences, like neglect and medical gaslighting, exacerbated by systemic inequities in health care systems like scarcity of funding, lack of clinical care, and denial or lack of research. Experiencing these biases as part of research and health care teams compounds these issues, and can contribute to further medical trauma as part of research teams.

**3. Lack of support to fully include patient partners**

Support to fully engage and include patient partners on teams may range from something as seemingly simple as the time of day at which meetings are hosted, to having a budget to pay upfront or to reimburse expenses to participate on the team (should be a given), compensation if patient partners wish to receive it (viewed as a best practice), training for patient partners and other team members, technology supports, salary for a person on the team or at the institute/hospital to support patient engagement, and more. While engaging patient partners may require more time and resources, not everyone engaging patient partners is fully aware of this. From our own experiences, we have seen the following:

- Meetings or conferences organized without flexibility in setting (e.g., outside of healthcare facilities), time, or options to catch up with a point person if the date/time does not work. Many options outside of traditional meetings or conferences that might work best for patient partners are often not offered (e.g., for meetings: separate calls, the opportunity to provide written or verbal feedback, etc.)
- Assuming that everyone on the team can process information and keep up the same way. Patient partners who live with cognitive issues or intellectual or developmental disabilities are at a disadvantage when meeting formats are hosted with heavy agendas that have an overabundance of information or without breaks.
- Pushing back on or not offering compensation (monetary or nonmonetary) to patient partners. Rationale ranges from not having considered compensation to conflict of interest that will impact objectivity and impartiality [23, 24].
- Expecting patient partners to work alongside senior academics without compensation commensurate with role expectations or support in understanding the complexities of working within an institution. For example, being a patient partner principal or co-principal investigator while being provided a gift card or paid minimum wage adds to the power imbalance.
- Failing to ‘do the work’ around reimbursement of expenses or compensation and passing the work off to patient partners (i.e., unfamiliar and tedious forms and processes, long expense reimbursement times that may have significant impact on their personal finances, etc.) [25].
- Lack of leadership support for meaningful inclusion, such as having team members understand patient partners’ conditions and offering support that individuals may need to do their work.
- No time dedicated to building relationships with patient partners, instead ‘hitting the ground running’ without fully knowing or understanding the dynamics of the research team.
- Assuming everyone has access to equipment, software, and expertise related to this work (not everyone has access to the Microsoft suite or to a technology support person!).
- Expecting patient partners to personally finance or find their own supports to attend meetings or conferences to which they have been invited.
- Terms of reference, codes of conduct or policies that are rooted in ableism (e.g., attendance requirements).

These examples may leave patient partners in need of asking for these (and other) supports. As people invited to the team, to a meeting, or to a conference, it is strange that these supports have not been anticipated, given thought, and taken care of so that the engagement can focus on the project. Many patient partners feel they are putting teams ‘out of their way’ when they ask for supports or other items that they had expected for their engagement but are not there. Often they will not speak up and ask given their fear of being seen as demanding, difficult, or even ungrateful.

**4. Lack of recognizing the vulnerability of patient partners**

Trained professionals who are on research or healthcare teams may not live with the condition under study or have experienced healthcare in the same ways that patient partners have (though sometimes they do and have, and do not always choose to disclose this). Professionals are often invited to the team because their skills, training and expertise are immediately recognized and valued. Patient partners usually have to work much harder to have their personal and professional skills and lived experiences valued, the latter of which are often not tangibly measured via a degree, diploma, or certificate. Not to mention that patient partners often re-live or re-experience very emotional or even traumatic parts of their lives for the sake of a project. This means being extremely vulnerable, sometimes in spaces with people with whom they are not very familiar. Examples we have seen here include:

- Being told that patient partners’ perspectives are biased and emotional, especially when professionals on the team do not share the same opinion. This can be further experienced as patient partners simply ‘whining’ about their experiences and being tuned out by other team members.
- Non-patient team members using ‘professionalism’ as an excuse to avoid connecting with other team members as individuals, not just as work colleagues. Expecting only patient partners to share personal information about themselves adds to their vulnerability.
- Meetings and interactions facilitated without creating a safe space or via a trauma informed lens. Some patient partners may be re-traumatized simply by being asked to meet at the hospital in which they had specific experiences.
- Using patient partners and their stories and their experiences to provide inspiration or data rather than meaningfully leveraging those stories and experiences to inform projects [26, 27].
- Having a single patient partner or not having a range of patient partners and their experiences on the team.
- A lack of recognition about how invested patient partners are in creating a better health care world, and a lack of honouring the importance of their stories and experiences and the fact that being engaged can sometimes trigger trauma.
- Patient partners feeling pressured to overshare components of their stories and experiences at the behest of the research team and feeling regret.

These experiences can further solidify an unspoken team hierarchy where only patient partners are expected to share very personal types of information and experiences. Using divisive terms such as ‘biased,’ ‘not objective,’ ‘subjective,’ and ‘emotional’ to describe patient partners’ experiences and expertise, while others on the team are ‘unbiased’ and ‘objective,’ is not appropriate or helpful. Patient partners bring their experiences (sometimes very painful or traumatic) to the team and expecting them to remain objective and unemotional is not right or fair. Being the only person on a team sharing raw experiences can be extremely alienating, especially if a safe space has not been created.

### Impact on patient partners (and others)

There is a power imbalance in research and quality improvement teams that include patient partners [10, 11]. Patient partners are invited into unknown or unfamiliar spaces in which they may be intimidated by the knowledge and expertise of others. Patient partners often feel concerned about the need to strike a balance between asking for supports and raising conflicting perspectives without appearing to be asking for too much and rocking the boat. The situations we have described contribute to widening this power imbalance and patient partners bear the brunt of this deepening divide.

The experiences we describe are unfortunately a little discussed part of patient engagement [28]. They leave mostly invisible impacts on patient partners: mental and physical exhaustion, worsened health, questions about the worth of engagement, and a feeling of failure (e.g., failed the team, failed patient communities, and failed overall to achieve personal goals or motivations). It may feel like the burden is on patient partners to educate a team on how to do engagement well and with that, a heavy responsibility to represent the entire patient community. It brings us right back to all the vulnerable health care situations we have experienced and reminds us of where we are in the hierarchy—at the bottom. It calls us to question if it is really worth doing this work so others do not experience the same as us.

Patient partners’ work in research and healthcare is very personal, and we can only deal with so much before it breaks us, our spirit and our desires to create change. These situations compound over time, especially if experienced by the same person or if patient partners exchange stories with other patient partners about similar experiences and feelings about them. Eventually, our fire goes out and the spark may not always be re-ignited. Some patient partners have suggested and created on-line support groups [29] and initiatives to provide peer support for negative patient engagement experiences.

So, what is the cost to society, to research and to healthcare, when we burn through patient partners? What do we collectively miss out on? These experiences do not just impact patient partners—they also affect others on the team. If patient partners do not feel supported, heard, or that they are equal to others on the team, there is a real possibility that they will leave an engagement or the engagement space altogether (other than for specific health purposes related to the conditions we or our loved ones are dealing with). Patient partners may not invest in developing the next generation of patient partners who will continue the momentum to change health care for others. When these spaces lose our lived expertise and skills, there is a potential for teams to gain a reputation for ‘using’ or not fully respecting patient partners when or if patient partners share these poor experiences with others in their communities.

### For reflection

We have had a number of positive patient engagement experiences, which may also help for reflection. These have been experiences where: we have been supported to share our experiences and thoughts openly and safely with the team; there has been more than one patient partner; there have been open and transparent communications, including what can or cannot be changed in a project; there have been a variety of options to engage (e.g., virtually, in-person); disability accommodations have been provided; and we have been offered compensation for our time, expertise and efforts. Further, we have felt like equal members of the team. It is these experiences that motivate us to share both our positive and negative experiences in hopes that others can learn from them.

We realize that engaging patients as partners is still relatively new for many people, and there is an opportunity to reflect on and learn from what does not work so well. While many individuals would rather not discuss these experiences, we feel compelled to share them so that we can grow as an engagement community. Without relying on or pressuring individual patient partners to vulnerably and painfully bring these experiences to attention, we have put these collective experiences out in the open.

### Take-away action for readers

We ask you to consider how and why these examples happen. How do you, in your own work related to patient engagement, accept feedback (if you are open to it) from patient partners and integrate it into your work? How can you ask more thoughtful questions of patient partners about their lived experiences? How can you create safer and more inclusive spaces for real and meaningful discussions? And how can you share your power and privilege with patient partners to move the field of patient engagement forward?

## Conclusion

Based on our experiences as patient partners in Canada, we present here a number of examples we have experienced where patient engagement has been ineffective, demoralizing and harmful. We provide four statements of experiences and anonymous examples to illustrate these: patient partners as a check mark, unconscious bias, lack of support to fully include patient partners, and lack of recognizing the vulnerability of patient partners. Our ask of readers is to reflect on these situations to see how they may best recognize these, learn from these, and strive to avoid these in their own work, so we can collectively move the field of patient engagement forward.

## Data Availability

Not applicable.
